# Travellers' malaria - 'one shoe does not fit all'

**DOI:** 10.1186/1475-2875-10-129

**Published:** 2011-05-17

**Authors:** Patricia Schlagenhauf, Marcel Hommel

**Affiliations:** 1 University of Zürich Centre for Travel Medicine, Hirschengraben 84, Zürich, Switzerland; 2 University of Liverpool, Liverpool, UK

## Abstract

Travellers' malaria is an exciting topic. It is a field in flux with evolving options for chemoprophylaxis, self-diagnosis, self-treatment, risk/strategy analyses and surveillance. Ideologies vary and experts differ but debate is needed and can bring change. The launch of a new thematic series in the *Malaria Journal *-- " Travellers' malaria " -- creates an ideal forum to bring together research papers, reviews, opinion papers and commentaries, and will hopefully stimulate debate.

## Editorial

This editorial highlights a new focus for the Malaria Journal namely "travellers' malaria". Malaria in travellers is a complex theme. Plasmodia stay plasmodia - but malaria in travellers often presents differently than the disease in semi-immune residents of endemic areas and this may result in missed or tardy diagnoses. Travellers are diverse: migrants, tourists, occupational travellers and refugee populations constitute an increasingly large and mobile segment of the world population. Each of these traveller types has individual needs and malaria risk profiles and one shoe does not fit all. This means that guidelines and recommendations are tailored to suit varying needs, leading to diversity in international guidelines on strategies, means and methods for monitoring, preventing, diagnosing and controlling disease. Malaria in migrants poses a formidable challenge in many industrial countries and several studies have shown an increased risk of malaria acquisition in travellers who return to their country of origin visiting friends and relatives (VFR). Almost all of these reports highlight the fact that those who need malaria prevention most are least likely to access pre-travel advice. Travel medicine needs to meet this challenge and find new ways to target such hard-to-reach, high-risk groups. Innovative means of communicating malaria risk and preventive strategies to the travel health provider, the travel industry and the traveller are key.

Surveillance of imported cases and the epidemiology of malaria at travellers' destinations are dynamic fields. Travel to and from endemic areas has resulted in imported malaria being frequently reported in malaria free countries. These data are captured to some extent by national authorities and in many industrialised countries, malaria is a notifiable disease but under-reporting is rampant and in many countries, official figures represent just the tip of the iceberg. Overall, official figures show a decline in imported cases in recent years but an increase in the proportion of malaria caused by *Plasmodium falciparum*. Imported malaria statistics from some popular traveller destinations such as India have shown a marked decline prompting some authorities to change their recommendations for prevention. There is dichotomy in guidelines worldwide particularly with regard to the use of stand-by self-treatment by travellers and debate and discussion on strategies is important. Surveillance networks, such as GeoSentinel [1], EuroTravNet [2] and TropNetEurop [3] have a key role in the surveillance of imported malaria, in identifying changing trends in malaria importation, in resistance patterns and in the changing profile of malaria risk at traveller destinations. These networks will have increasing importance in capturing data on migrant malaria.

Although the topic of travellers' malaria is not new to Malaria Journal - 25 relevant papers published over the past three years - this new Thematic Series will further emphasize the importance of the subject and provide a forum for debate. Travellers' malaria is mobile and global.
